# Supplementary material for: Using Macrophage Polarization in Human Platelet Lysate to Test the Immunomodulatory Potential of Cells for Clinical Use
Source: Biomedicines. 2024 Apr 9;12(4):833. doi: 10.3390/biomedicines12040833 (PMC11048141; doi:10.3390/biomedicines12040833)
Supplement: Supplementary file 1 [file biomedicines-12-00833-s001.zip › biomedicines-2917677-supplementary.pdf]

Supplementary Information

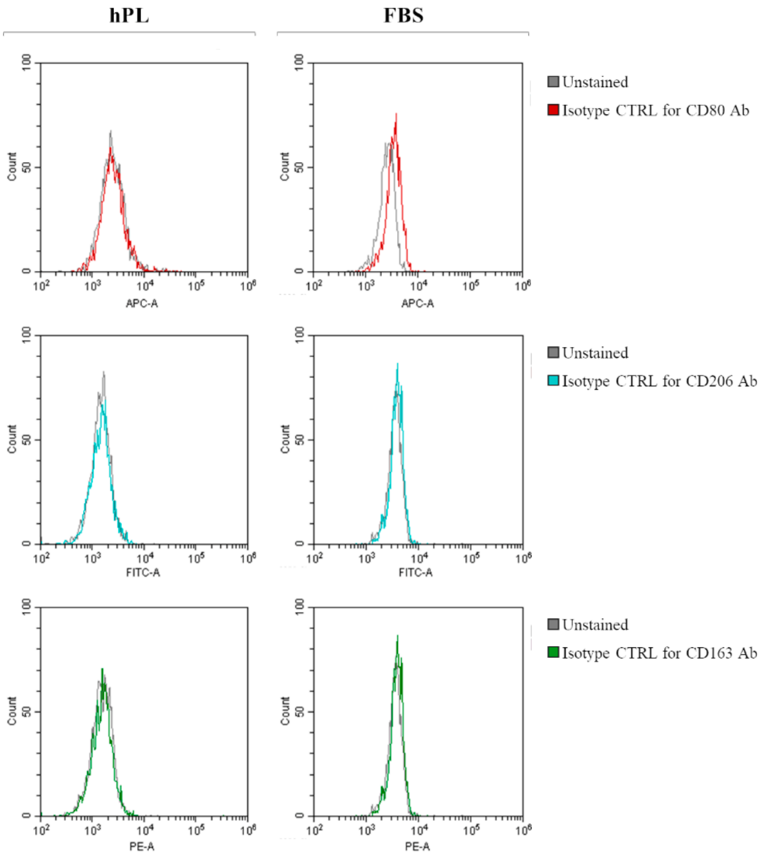

Supplementary Figure S1. Isotype controls for CD80, CD206, and CD163 antibodies.

hPL

	M1	M2a	M2c
IL1α	1.9	1.3	1.3
IL1β	3.7	1.2	1.2
IL8	4.3	0.6	2.7
TNFα	954.3	1.0	2.4
CCL2	3.8	0.7	1.6
CCL3	480.0	1.4	1.3
CXCL9	69.4	1.2	1.1
sCD163	1.3	0.9	1.0
IL4	1.9	325.4	1.6
IL13	1.7	41.6	1.3
CCL18	2.6	17.0	3.0
CCL22	0.4	2.9	0.9
IL10	3.9	1.2	89.8
MMP7	2.5	0.9	3.0
TIMP1	1.1	1.1	1.4

FBS

	M1	M2a	M2c
IL1α	1.7	1.5	1.5
IL1β	3.2	1.7	1.5
IL8	2.0	0.9	1.2
TNFα	335.8	1.4	2.2
CCL2	0.9	0.7	1.1
CCL3	72.2	1.8	1.3
CXCL9	121.8	1.4	1.3
sCD163	1.9	1.2	1.6
IL4	1.8	484.9	1.4
IL13	1.6	42.4	1.4
CCL18	3.4	15.3	6.7
CCL22	0.2	31.5	0.7
IL10	37.7	1.0	136.9
MMP7	1.1	1.5	2.2
TIMP1	1.3	0.9	1.7

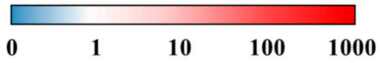

Supplementary Figure S2. Secretory profiles of polarized macrophages cultured in hPL or FBS expressed as average fold increase (FI) vs. unpolarized macrophages (M0). Heatmaps show FI for each marker in color scale ranging from blue (FI < 1) to red (FI > 1). Data were obtained from independent experiments conducted with three different batches of macrophages.

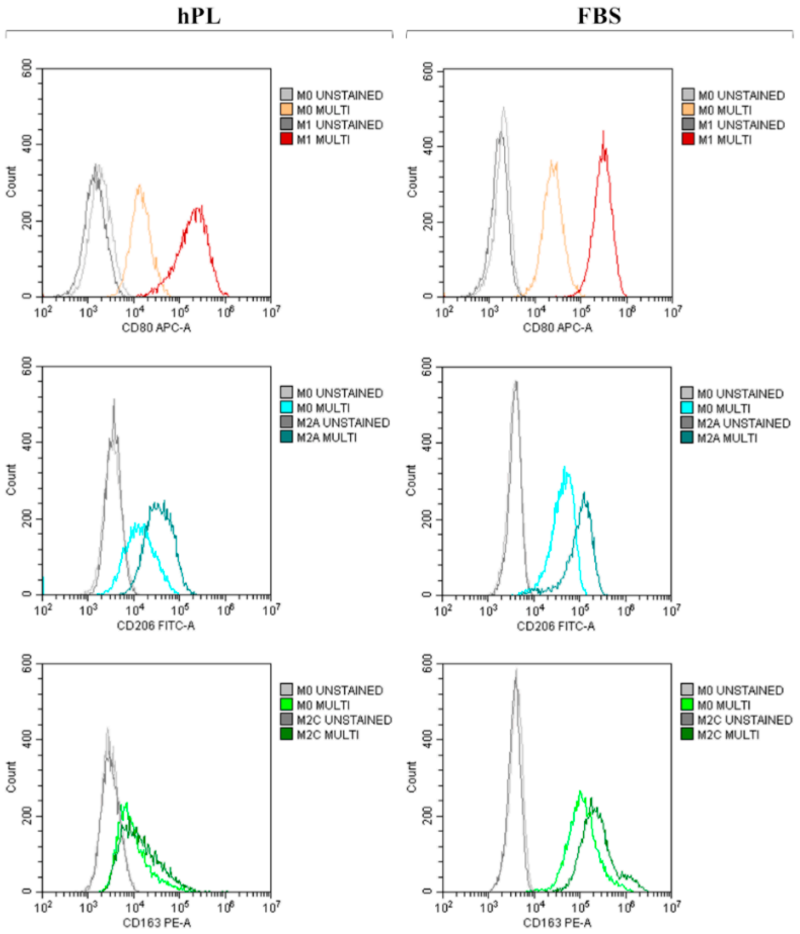

Supplementary Figure S3. Representative overlay histograms showing the expressions of phenotype-specific markers in M0 macrophages and polarized macrophages cultured either in hPL or in FBS. The unstained samples are represented as grey curves.

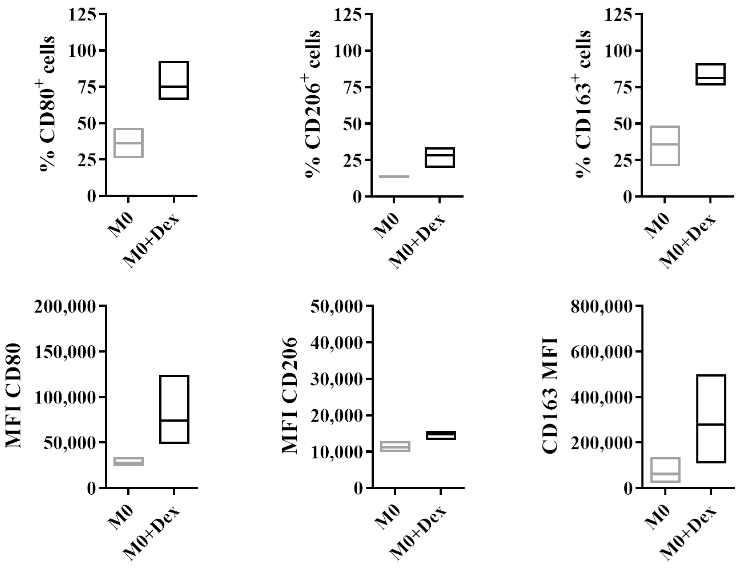

Supplementary Figure S4. Cell surface marker expressions in unpolarized macrophages cultured in hPL to compare the phenotype of the macrophages alone (M0) with that of the macrophages treated with dexamethasone (M0+Dex). Dexamethasone 10^{-7} M (Sigma-Aldrich), a drug commonly used to control OA-related inflammation, was used to polarize monocytes towards an anti-inflammatory phenotype to obtain a control group of the co-culture model. The min-to-max box plots (with line at mean) show data expressed both as the percentage of positive cells and as the Mean Fluorescence Intensity (MFI) of the cell population.

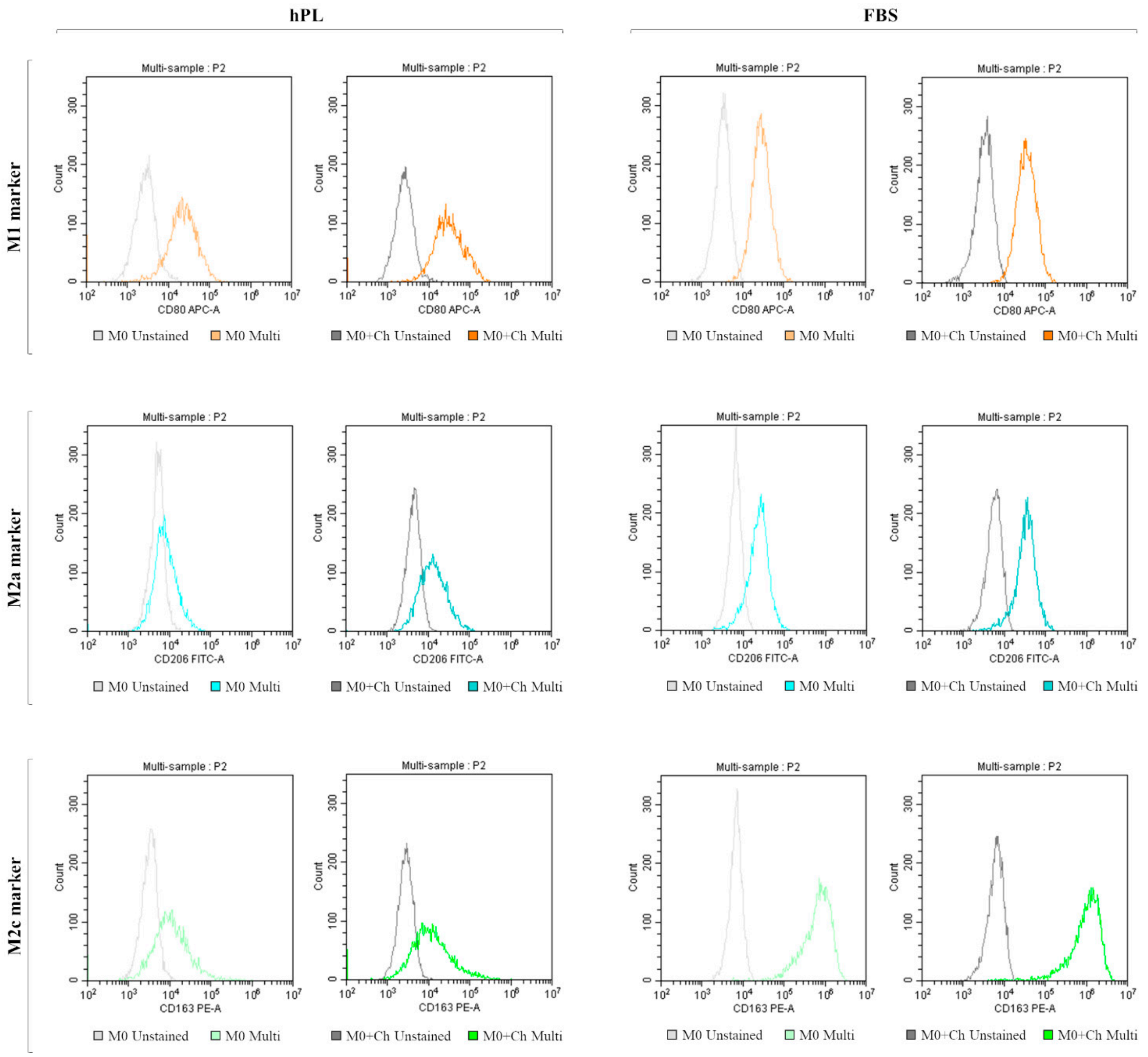

Supplementary Figure S5. Representative overlay histograms showing expressions of phenotype-specific markers in M0 macrophages alone and co-cultured with chondrocytes either in hPL or in FBS. Unstained samples are represented as grey curves.
